# Digital phenotyping for mental health monitoring and personalized prediction in people with diabetes: a Python-based scoping review

**DOI:** 10.3389/fdgth.2026.2005262

**Published:** 2026-10-28

**Authors:** Wenhui Hou, Wei Sun, Haochen Dai, He Yang, Guodong Xu, Xuefang Xie, Fengzhen Bai, Dong Xie

**Affiliations:** 1 School of Nursing, Changchun University of Chinese Medicine, Changchun, Jilin, China; 2 School of Public Health, Jilin University, Changchun, Jilin, China

**Keywords:** diabetes mellitus, digital phenotyping, machine learning, mental health, personalized prediction, scoping review

## Abstract

**Background:**

Diabetes frequently co-occurs with depression, anxiety, and diabetes-related distress, and psychological status directly influences glycaemic control and the risk of complications. Traditional mental health assessment relies on subjective scales. With the growth of digital health, digital phenotyping offers a new paradigm for real-time, objective mental health monitoring and prediction. However, the pathways through which these technologies perform monitoring and prediction remain unclear.

**Objective:**

This scoping review aimed to systematically map the application pathways of digital phenotyping for mental health monitoring and prediction in people with diabetes, and to use machine learning-assisted text mining to analyse the main research themes and hotspots, thereby informing the future development of individualised risk prediction models and monitoring systems.

**Methods:**

A scoping review search was conducted in six English databases (PubMed, Embase, CINAHL, Web of Science, Scopus, and the Cochrane Library) and four Chinese databases (CNKI, Wanfang, VIP, and China Biomedical Literature Database[CBM]), covering studies published from database inception to 10 September 2026. The search strategy was built around terms related to diabetes, digital phenotyping, and mental health. Machine learning-assisted literature mining was used to identify thematic patterns in the included studies. The study selection process and overall workflow followed the PRISMA extension for scoping reviews (PRISMA-ScR).

**Results:**

A total of 2,968 records were retrieved. After screening, 10 studies were included. The evidence indicated that digital phenotyping mainly enables real-time monitoring of mental health in people with diabetes through signals such as continuous glucose monitoring (CGM), passive smartphone sensing, voice, and ecological momentary assessment (EMA). Machine learning methods were widely used for risk prediction modelling, but most studies built models at the group level, with only a few adopting *n*-of-1 analysis or subgroup prediction to achieve individualised risk prediction. External validation of models was generally lacking, and no intervention studies were identified.

**Conclusions:**

Preliminary evidence suggests that digital phenotyping has exploratory potential for mental health monitoring and prediction in people with diabetes, but research that genuinely achieves individualised risk prediction remains limited. Closed-loop monitoring-intervention design and multicentre external validation are the main directions for future research.

**Systematic Review Registration:**

https://osf. io/6wsaj/.

## 1 Introduction

Diabetes is a chronic metabolic disease characterised primarily by hyperglycaemia, and poor diabetes control leads to various complications (1). In recent years, multiple studies have demonstrated a bidirectional relationship between diabetes and mental health (2, 3). People with diabetes face various chronic stressors, and elevated cortisol levels are associated with increased levels of anxiety and depression (4). In addition, the burden of diabetes management can trigger or exacerbate mental health problems, adversely affecting glycaemic control, treatment adherence, and quality of life (5). Although these mental health conditions and diabetes form a reciprocal cycle, mental health support is often neglected or marginalised in the routine care of people with diabetes (6). Traditional mental health assessment relies mainly on self-report scales and clinical interviews. Although these are highly standardised, they reflect retrospective psychological states at discrete time points, making it difficult to capture real-time fluctuations in daily life, and they are susceptible to recall bias. With the development of digital health, digital phenotyping-the collection of physiological, behavioural, or voice signals via smartphones, wearables, and continuous glucose monitoring (CGM), and including both passive sensing and active ecological momentary assessment (EMA)-offers a new pathway for real-time, objective, and ecologically valid mental health monitoring and prediction. A systematic review noted that digital phenotyping has been used to monitor various psychiatric disorders, but the methodological approaches are highly heterogeneous (7). Notably, research targeting the specific population of people with diabetes remains scarce. A feasibility study showed that smartphone-based digital phenotyping has high acceptability among people with type 2 diabetes (8). Moreover, the coordinated integration of glucose monitoring, self-care behaviours, and mental health data is considered an important direction for achieving precision monitoring in diabetes (9). However, integrated research applying digital phenotyping simultaneously to diabetes management and mental health monitoring remains limited. In view of this, this scoping review aimed to systematically map the research evidence on digital phenotyping for mental health monitoring and prediction in people with diabetes, to identify signal types and monitoring indicators, and to apply machine learning-assisted text mining to analyse thematic distribution, research hotspots, and emerging knowledge structures, thereby providing a reference for future theory-based monitoring and prediction models.

## 2 Methods

### 2.1 Study design

This study was conducted as a scoping review, and its design and reporting followed the PRISMA extension for scoping reviews (PRISMA-ScR) (10). The review protocol was registered on the Open Science Framework (https://osf. io/6wsaj/). The registration was completed after screening had begun; therefore, this review is not a strictly prospective registration. This limitation does not methodologically affect the transparency or reproducibility of the review. The full search strategy is provided in Supplementary Material S1. Consistent with scoping review methodology, this review aimed to map the breadth of evidence on digital phenotyping for mental health monitoring and individualised risk prediction in people with diabetes and to clarify the conceptual structure of the field. Python-based text mining methods (LDA topic modelling, BERT semantic clustering, and keyword co-occurrence analysis) were used to mine the included literature.

### 2.2 Research questions

To clarify the research questions and define the scope of the review, this review was guided by the PCC (Population, Concept, Context) framework and focused on the following core questions: (1) What are the main application contexts, research foci, and population characteristics of digital phenotyping in mental health monitoring for people with diabetes? (2) What types of digital phenotyping have been used in existing studies? (3) What associations exist between the mental health outcomes identified by digital phenotyping and monitoring indicators? What machine learning methods have been used for risk prediction, and which studies have genuinely achieved individualised risk prediction? (4) What are the thematic distribution, hotspots, and knowledge structure of this field, and what research gaps exist?

### 2.3 Search strategy

Following the PRISMA-ScR guidelines and the framework of Arksey and O'Malley (11), a comprehensive literature search was conducted to identify studies on digital phenotyping for mental health monitoring and individualised risk prediction in people with diabetes. The following electronic databases were searched: six English databases (PubMed, Embase, CINAHL, Web of Science, Scopus, and the Cochrane Library) and four Chinese databases (CNKI, Wanfang, VIP, and the CBM). The search covered studies published from database inception to 10 September 2026. The search strategy combined controlled vocabulary and free-text terms, built around terms related to diabetes, digital phenotyping, and mental health. Boolean operators (AND, OR), truncation (*), and field tags (e.g., [Title/Abstract]) were applied as appropriate to ensure comprehensiveness. The search was limited to publications in English or Chinese; this limitation may have introduced language-related selection bias. The complete search strategies for the 10 databases are provided in Supplementary Material S1.

### 2.4 Inclusion and exclusion criteria

#### 2.4.1 Inclusion criteria

Studies were included if they met the following criteria: (1) The study population comprised people with diabetes (type 1, type 2, or unclassified), with no age restriction; (2) The study explicitly examined the impact of digital phenotyping on mental health monitoring or individualised risk prediction in people with diabetes and included at least one of the following four types of digital phenotyping signals: ① Continuous glucose monitoring (CGM) signals, including glycaemic indicators collected by intermittently scanned CGM (isCGM) or real-time CGM (e.g., time in range, glycaemic variability, hypoglycaemic events); ② Passive smartphone sensing signals, including accelerometer, GPS, screen time, call logs, and ambient light; ③ Voice signals, including acoustic features and voice biomarkers obtained through voice-capture devices or smartphones; ④ Ecological momentary assessment (EMA) signals, which, although actively reported, were included within the scope of digital phenotyping because of their real-time, repeated collection in natural environments; (3) The study was an original empirical study, including cross-sectional, cohort, longitudinal observational, or mixed-methods studies; and (4) The publication was written in Chinese or English. The PCC mapping and inclusion rationale for each included study are provided in Supplementary Material S2.

#### 2.4.2 Exclusion criteria

Studies meeting any of the following criteria were excluded: (1) Animal studies, case reports, reviews, commentaries, or other non-original articles; (2) Studies not focused on the impact of digital phenotyping on mental health in people with diabetes; (3) Duplicate publications, studies with incomplete data, or studies from which key information could not be extracted; (4) Studies for which the full text was unavailable; (5) Studies using only traditional scales without digital phenotyping signal collection; and (6) Studies focusing only on physiological indicators of diabetes without mental health outcomes.

### 2.5 Study selection

All retrieved records were imported into EndNote X9 for duplicate removal. Two reviewers, both trained in evidence-based research methods, independently screened titles and abstracts according to the pre-specified inclusion and exclusion criteria. Full-text articles considered potentially eligible were then retrieved and assessed for inclusion. Disagreements during screening were resolved through discussion with a third reviewer until consensus was reached. The study selection process and overall workflow followed PRISMA-ScR and are presented as a flow diagram, recording the number of records and reasons for exclusion at the identification, screening, eligibility, and inclusion stages.

### 2.6 Data extraction

Data extraction included the following items: author information, year of publication, country of study, study design, follow-up duration, sample size, participant characteristics (diabetes type), type of digital phenotyping signal(device type, signal type), mental health outcomes (outcome measures, measurement tools), machine learning methods, and main findings. All extracted data were systematically organised using a standardised data extraction form. Because the primary purpose of a scoping review is evidence mapping rather than pooled effect estimation, no formal risk of bias assessment was performed; however, descriptive methodological indicators such as study design and sample size were extracted to aid interpretation of evidence quality.

### 2.7 Data analysis

#### 2.7.1 Text preprocessing

Through text mining, high-frequency keywords and core concepts were identified from the 10 included studies. After PDF-to-text conversion, the raw text was preprocessed using Python cleaning scripts, including removal of reference lists, author affiliations, journal headers and footers, figure and table titles, table data rows, abbreviation footnotes, and in-text citation markers. The cleaned corpus consisted of 1,354 valid sentences, containing 21,301 words and 3,307 unique vocabulary items, with an average of 15.73 words per sentence. Tokenisation was performed using the NLTK library; the stopword list used the NLTK standard English stopword list, supplemented with custom stopwords related to manuscript structure (e.g., study, result, method). Lemmatisation was performed using WordNetLemmatizer. In LDA modelling, the filter_extremes function was used to remove vocabulary that occurred too infrequently (no_below = 2) or too frequently (no_above = 0.9). For biomedical terms (e.g., CGM, EMA, HbA1c, PAID), the original forms were retained during the keyword matching stage; during text preprocessing, some high-frequency abbreviations were uniformly converted to lowercase, but no additional synonym mapping or terminology standardisation was performed. Negation words were not specially handled in this study; all sentences were tokenised and word frequencies were counted in their original word order, without negation detection or semantic reversal. The abbreviation handling rules are provided in Supplementary Material S3.

#### 2.7.2 Topic modeling using LDA

Through systematic comparison of coherence scores across models (*K*=2-10), the coherence score was highest at *K*=3 (0.4501), and *K*=6 was selected as a balance between coherence, topic stability, and interpretability. Although *K*=3 and *K*=4 had slightly higher coherence scores (0.4501 and 0.4427, respectively), they produced fewer topics that could not capture the multiple dimensions of the research questions (CGM, mental health, machine learning, voice features, and EMA). *K* = 6 provided six interpretable topics that align closely with the research questions, with a coherence score of 0.4231 and the highest topic stability among all tested *K* values (mean Jaccard similarity = 0.3354). *K* = 7 was also qualitatively evaluated, but its topic stability was slightly lower (Jaccard = 0.3332). In addition, the stability of the *K* = 6 topics was assessed across five independent runs (random seeds 42, 1, 2, 3, and 4), showing moderate stability (mean Jaccard similarity = 0.3354, range 0.1765–0.6667).

#### 2.7.3 BERT-based semantic clustering

To complement word-level topic modelling, BERT-based semantic clustering was performed using the pre-trained all-MiniLM-L6-v2 model. The silhouette score was highest at *K* = 2 (0.0656), indicating that *K* = 2 was the independently optimal number of clusters for BERT. However, to facilitate direct comparison with the LDA topic model (*K* = 6), *K* = 6 was also selected for BERT clustering. This choice was made for comparison purposes, rather than because *K* = 6 was the independently optimal number of clusters. The silhouette scores were low across all *K* values (all <0.07), indicating that no single *K* value provided clearly superior cluster separation. The semantic clusters and corresponding word clouds are presented.

#### 2.7.4 Keyword co-occurrence network analysis

Using a predefined keyword dictionary covering four conceptual categories—digital phenotyping signals (Signal), mental health (MentalHealth), machine learning (ML), and glucose (Glucose)—a domain-specific keyword co-occurrence network was constructed. Co-occurrence frequency was calculated by counting the number of times different conceptual categories appeared together in the same sentence. The complete keyword dictionary is provided in Supplementary Material S3.

#### 2.7.5 Co-occurrence pattern analysis

In the included studies, the same keyword dictionary was used to perform sentence-level classification of text related to digital phenotyping signals and mental health outcomes. The co-occurrence frequencies of different conceptual categories within the same text unit were then calculated, and a keyword co-occurrence matrix and co-occurrence frequency bar chart were constructed to identify the strength and structural characteristics of conceptual associations.

#### 2.7.6 Software and version control

Text mining and machine learning analyses were performed primarily in a Python 3.11 environment. Libraries including pandas, nltk, scikit-learn, gensim, sentence-transformers, networkx, and seaborn were used for text preprocessing, topic modelling, semantic representation, clustering analysis, and visualisation. To ensure reproducibility, the raw data, cleaned text, analysis scripts, and result files are available from the corresponding author upon reasonable request.

#### 2.7.7 Quality control and manual validation

To improve the reliability of the analysis results, irrelevant information and anomalous words were manually reviewed and removed during the text preprocessing stage. After topic modelling and clustering analysis, two researchers reviewed the results in conjunction with the original study content and, where necessary, performed semantic refinement to ensure the accuracy of topic classification and pathway identification. Inter-rater agreement (e.g., Cohen's kappa) was not measured in this study because the qualitative review of topics and clusters involved interpretive judgement rather than a standardised classification task, and kappa values may not accurately reflect the degree of semantic disagreement.

#### 2.7.8 Rationale for model selection

This study adopted an integrated analytical approach combining LDA, BERT-based semantic representation, clustering, and co-occurrence analysis. LDA and BERT were selected because they provide complementary perspectives on the text corpus. LDA identifies recurring themes based on word distribution patterns, whereas BERT-based semantic representation captures contextual similarity between passages that may use different terminology. Clustering was used to organise semantically related passages, and co-occurrence analysis quantified the relationships among digital phenotyping signals, mental health outcomes, and machine learning methods. These analyses were not used to estimate intervention effects or establish causality; rather, they complemented manual synthesis by revealing corpus-wide patterns, neglected themes, and cross-study semantic relationships. All algorithm-derived topics and clusters were independently reviewed by two researchers against the source literature before being interpreted and incorporated into the final thematic framework.

## 3 Results

A total of 2,968 records were initially retrieved, and 2,163 remained after duplicate removal. After title/abstract screening, 1,074 records were excluded, mainly because of inappropriate study type (*n* = 805) or lack of focus on digital phenotyping (Figure 1).

**Figure 1:**
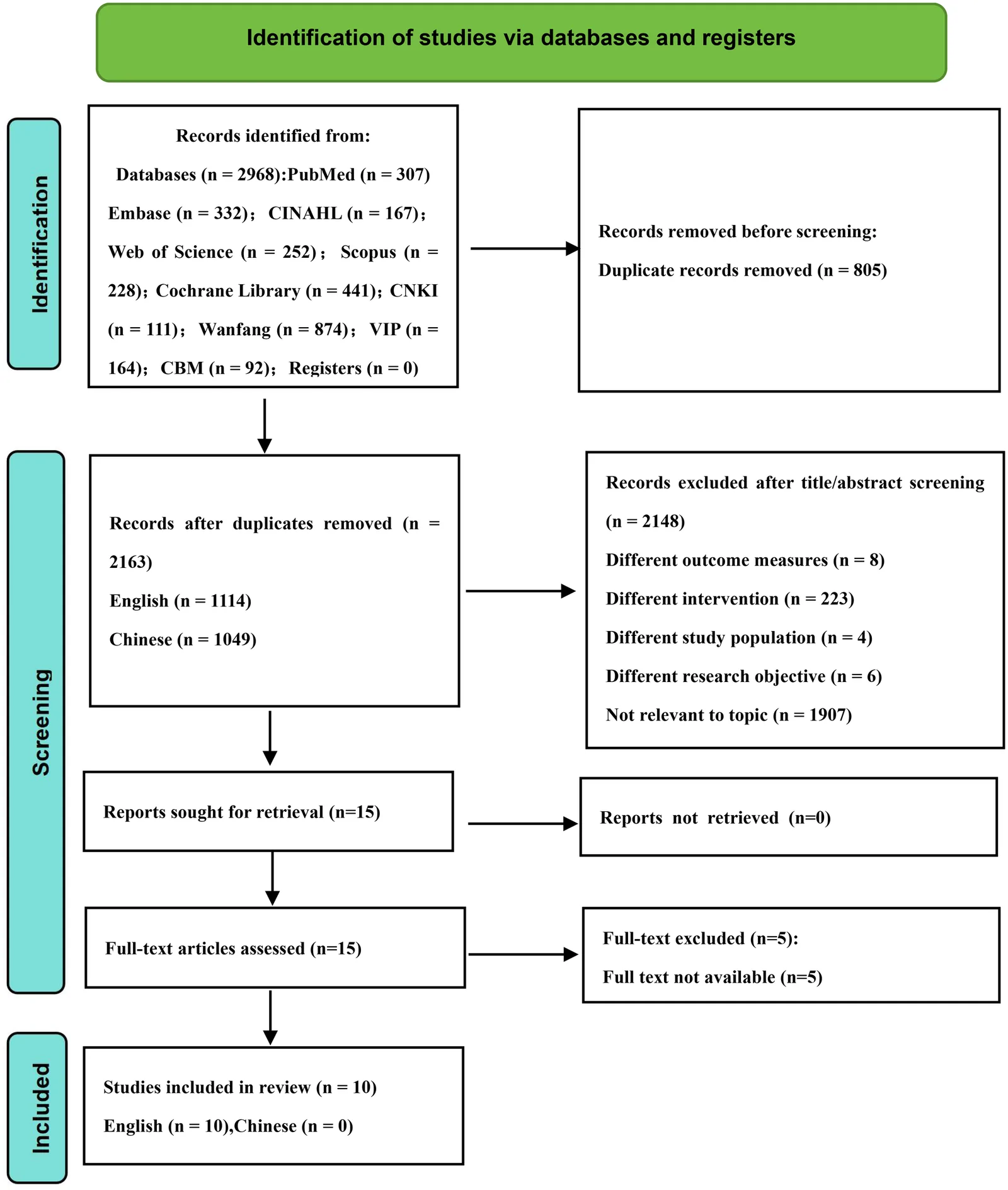
PRISMA flow diagram showing the screening and selection process of included studies.

### 3.1 Characteristics of included studies

A total of 2,968 records were retrieved. After duplicate removal, title/abstract screening, and full-text assessment, 10 studies were ultimately included (Tables 1, 2). Among the 10 included studies, only 3 genuinely achieved individualised risk prediction: Ehrmann et al. (15) and Hermanns et al. (16) used *n*-of-1 analysis to build separate glucose-psychological state association models for each patient, while Lee et al. (19) used a Gaussian mixture model to identify four subgroups of glycaemic patterns, which constitutes subgroup prediction. The remaining 7 studies were based on group-level modelling or association analysis; although some studies (12, 13, 17) used individual-level data to train machine learning models, the models themselves were group-level models and did not constitute individualised risk prediction in the strict sense. The included studies were published between 2019 and 2026, and the study locations covered Japan, South Korea, Germany, Ireland, Luxembourg, the United States, and India. The study designs were mainly cross-sectional and observational, with a few longitudinal studies (EMA lasting 8–60 days). Sample sizes varied widely, ranging from 47 to 142,432, indicating high heterogeneity in study design, population size, and validation strategies. The included studies mainly involved type 1 and type 2 diabetes, with a predominance of young and middle-aged adults and few studies in older adults with diabetes. Digital phenotyping signals mainly included passive smartphone sensing, wearable devices, continuous glucose monitoring (CGM), and voice.

**Table 1:** Basic characteristics of the included studies (*n* = 10).

Reference	Country	Study design	Total sample size	Diabetes type	Digital phenotyping signal	Mental health outcome	Machine learning method	Main findings
Sarda et al. (12)	India	Cross-sectional observational	47	Type 1 + Type 2	Smartphone (accelerometer, GPS, ambient light, call logs)	Depression (PHQ-9)	SVM, decision tree, random forest, AdaBoost, XGBoost	XGBoost and random forest performed best (test accuracy 81.1%,80.0%); depressive symptoms were associated with lower social contact and lower daytime activity
Yu et al. (13)	USA	Retrospective case-control	142,432	Type 1 + Type 2	Glucose meter, app interaction, coaching interaction	Mental health risk (prescription/claims data)	LightGBM ensemble (10 models) + SHAP	Model AUC >0.65 across 3 test sets; SHAP identified race, sex, and emotional state during blood glucose checks as important signals
Hohendorff et al. (14)	Poland	Observational cohort	77	Type 1	isCGM (FreeStyle Libre)	Fear of hypoglycemia (HFS-II)	Correlation analysis, multiple regression	Higher scanning frequency was associated with better glycemic indicators and lower fear of hypoglycemia (HFS-II *r* = −0.25)
Ehrmann et al. (15)	Germany	Prospective observational (17-day EMA + 3-month follow-up)	379	Type 1 + Type 2	CGM + EMA	Diabetes distress (PAID), depression (CES-D)	Bayesian linear mixed-effects model + *N*-of-1 analysis	Subjective perception of glucose had a stronger impact on diabetes distress than objective CGM indicators; individualized drivers influenced mental health outcomes at 3 months
Hermanns et al. (16)	Germany	Longitudinal observational (8-day EMA)	371	Type 1 + Type 2	CGM + EMA	Depression (CES-D), diabetes distress (PAID)	Linear mixed-effects model + *N*-of-1 analysis	Eight symptoms were significantly associated with glucose; depression and diabetes distress significantly increased symptom scores (up to 12%)
Sakane et al. (17)	Japan	Cross-sectional	259	Type 1	CGM (FreeStyle Libre)	Diabetes distress (PAID)	Random forest, SVM, Naive Bayes, neural network, k-NN, XGBoost, LightGBM, and 10 algorithms in total	Neural network model performed best (AUC = 0.728, accuracy = 0.744); LBGI was the most important predictive feature
McInerney et al. (18)	Ireland	Longitudinal observational (2 months)	61	Type 2	Smartphone (accelerometer, GPS, EMA)	Daily mood (happiness, sadness, stress, anger)	Dynamic structural equation model (DSEM)	Sleep quality and physical activity were significantly associated with daily mood; the mood-behavior associations differed between patients with type 2 diabetes and healthy individuals
Lee et al. (19)	USA	Cross-sectional	137	Type 2	CGM (FreeStyle Libre Pro)	Diabetes distress (DDS-17)	Gaussian mixture model (GMM) clustering	Four glycemic patterns were identified; the sustained hyperglycemia group had the highest diabetes distress (mean = 2.37)
Bour et al. (20)	Luxembourg	Cross-sectional	679	Type 1 + Type 2	Voice (DisVoice)	Stress, diabetes distress (PAID)	Multivariate ordinal logistic regression	Stress was associated with 25 voice features; psychological distress was associated with 18 features; no overlap in voice patterns between the two states
Kim et al. (21)	South Korea	Cross-sectional	104	Type 1	CGM (Guardian Connect, Dexcom, FreeStyle Libre)	Stigma (DSAS-1), anxiety (GAD-7), depression (PHQ-9), diabetes distress (DDS)	Linear regression, logistic regression	Stigma was significantly associated with TBR <3.0 mmol/L; stigma was significantly associated with anxiety (OR = 1.086)

CGM, continuous glucose monitoring; EMA, ecological momentary assessment; isCGM, intermittently scanned continuous glucose monitoring; PAID, Problem Areas in Diabetes; DDS, Diabetes Distress Scale; PHQ-9, Patient Health Questionnaire-9; GAD-7, Generalized Anxiety Disorder-7; CES-D, Center for Epidemiological Studies Depression Scale; HFS-II, Hypoglycemia Fear Survey-II; DSAS-1, Type 1 Diabetes Stigma Assessment Scale; LBGI, Low Blood Glucose Index; SHAP, SHapley Additive exPlanations; AUC, area under the receiver operating characteristic curve; TBR, time below range; SVM, support vector machine; DSEM, dynamic structural equation model; GMM, Gaussian mixture model.

**Table 2:** Summary of population characteristics of the included studies (*n* = 10).

Study	Country	*N*	Diabetes type	Age (mean ± SD, or range)	Sex (M/F)
Sarda et al. (12)	India	47	Type 1 + type 2	Mean 35 ± 12 years; 60% aged 21–40	29 M/17 F
Yu et al. (13)	USA	142,432	Type 1 + type 2	Mean 54.8 ± 12.4 years	73,147 M (51.6%)/68,968 F (48.4%)/317 other (0.22%)
Hohendorff et al. (14)	Poland	77	Type 1	Mean 34.1 ± 10.2 years	19 M/58 F
Ehrmann et al. (15)	Germany	379	Type 1 + type 2	Type 1:39.1 ± 12.7 years; Type 2:52.8 ± 9.7 years	Overall 191 M (41.5%)/188 F (49.6%)
Hermanns et al. (16)	Germany	371	Type 1 + type 2	Type 1:38.7 ± 12.7 years; Type 2:53.3 ± 9.4 years	Type 1:80 M (41.7%)/112 F (58.3%); Type 2:105 M (58.7%)/74 F (41.3%)
Sakane et al. (17)	Japan	259	Type 1	Mean 48.9 ± 15.4 years	97 M (37.5%)/162 F (62.5%)
McInerney et al. (18)	Ireland	61	Type 2 (*n* = 32) + non-T2D controls (*n* = 29)	Median age category 50–54 years (both groups); no exact mean given	T2D:16 M/16 F; non-T2D:8 M/21 F
Lee et al. (19)	USA	137	Type 2	Mean 57.3 ± 12.9 years	62 M (45.1%)/75 F (54.9%)
Bour et al. (20)	Luxembourg	679	Type 1 + type 2	Males 44.2 ± 16.8 years; Females 45.3 ± 15.3 years	298 M/381 F
Kim et al. (21)	South Korea	104	Type 1	Mean 48.3 ± 17.1 years	41 M (39.4%)/63 F (60.6%)

### 3.2 Distribution of high-frequency keywords

Analysis of high-frequency keywords (Figure 2) showed that the most frequent terms were, in order: diabetes (approximately 529 times), data (approximately 337 times), study (approximately 256 times), distress (approximately 247 times), glucose (approximately 246 times), symptom (approximately 198 times), participant (approximately 178 times), type (approximately 140 times)，people (approximately 140 times) and health (approximately 123 times). These results indicate that, in the current corpus, the existing literature mainly focuses on diabetes management, glucose monitoring, and diabetes-related distress. Notably, distress and symptom ranked fourth and sixth, respectively, reflecting that diabetes-related distress has become an important issue in this field.

**Figure 2:**
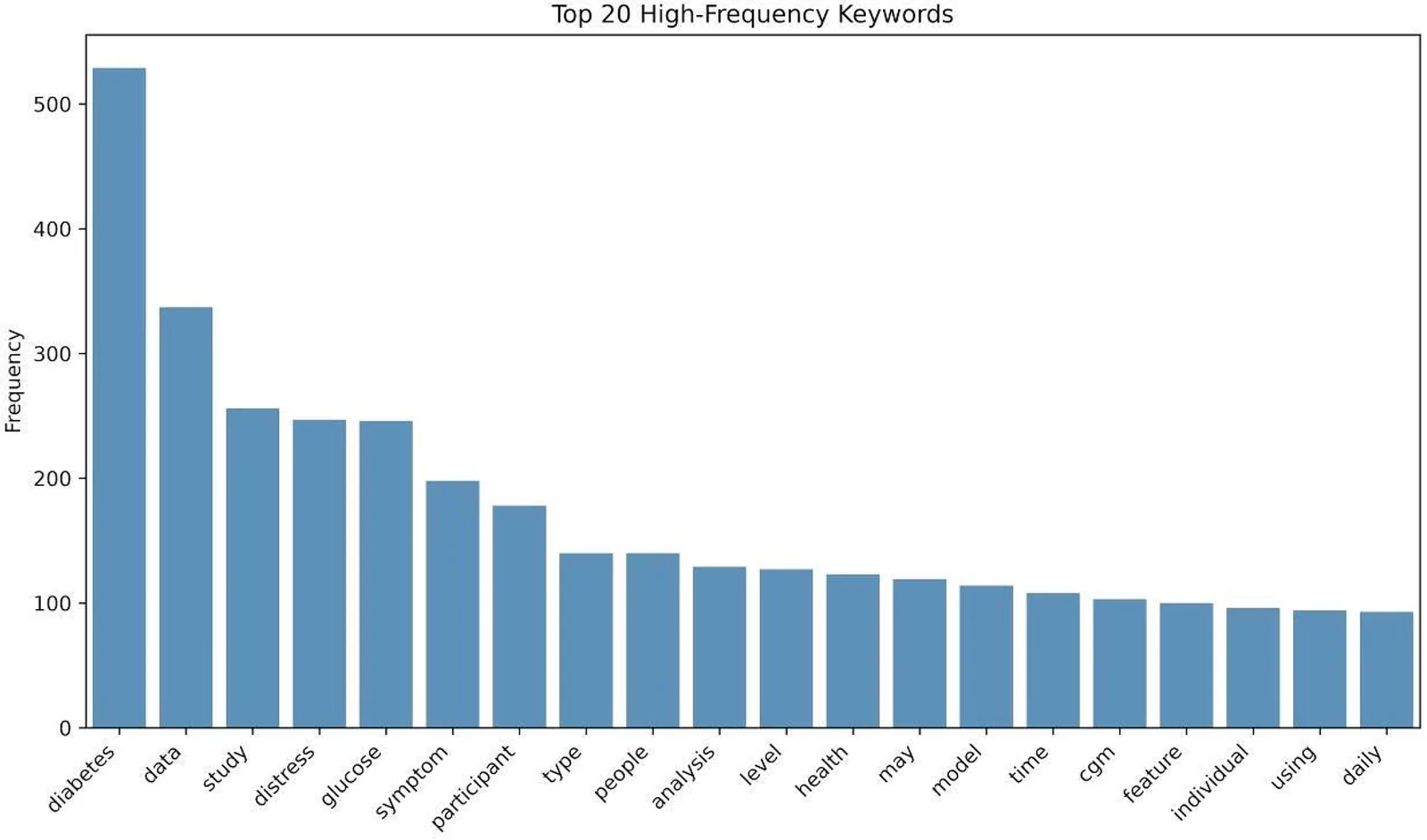
Bar chart of the top 20 high-frequency keywords in the included corpus.

### 3.3 LDA topic structure analysis

LDA topic modelling identified six topics from the 1,354-sentence corpus (Table 3). The number of topics, *K* = 6, was selected as a balance between coherence, topic stability, and interpretability (see Sec. 2.7.2 and Figure 3). The distribution of the Top 20 high-frequency words across the six topics is shown in Figure 4.

**Table 3:** Six topics identified by LDA topic modeling.

Topic	Topic Name	Keywords	Meaning
0	Diabetes symptoms and depression	Diabetes, symptom, glucose, distress, level, type	Diabetes symptoms (glucose, distress) and their association with depression
1	Stigma and psychological scales	Diabetes, study, type, stigma, score, higher, participant	Diabetes stigma and psychological measurement scales
2	Sleep, mood, and physical activity	Data, people, mood, sleep, study, effect, activity, ema	Sleep, mood, and physical activity in daily life
3	Machine learning and passive sensing	Data, participant, health, set, event, mental, test, signal, passive	Machine learning modelling and passive sensing signals
4	Machine learning and model evaluation	Data, variable, model, instance, sensing, value, random, recall	Machine learning feature selection and model evaluation
5	Diabetes distress and glycaemic patterns	Diabetes, distress, glucose, feature, stress, cluster, glycemic, CGM, vocal	Diabetes distress, glycaemic patterns, and CGM indicators

LDA topic modelling identified 6 topics from the 1,354-sentence corpus. Keywords are sorted by weight, and the top 6 words with the highest weight in each topic are presented. EMA, ecological momentary assessment; CGM, continuous glucose monitoring.

**Figure 3:**
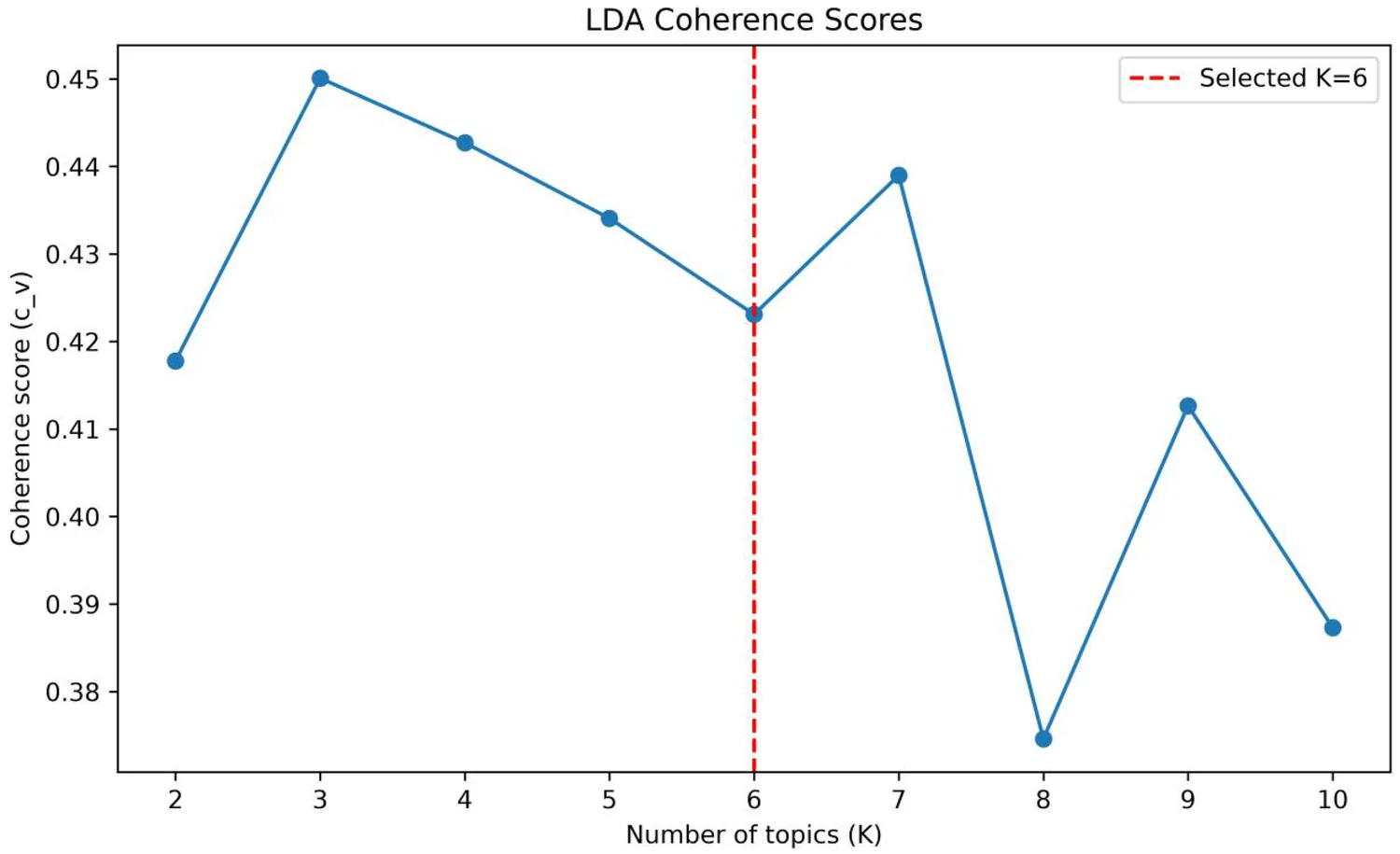
LDA coherence scores for different topic numbers; *K* = 6 was selected as the optimal topic number.

**Figure 4:**
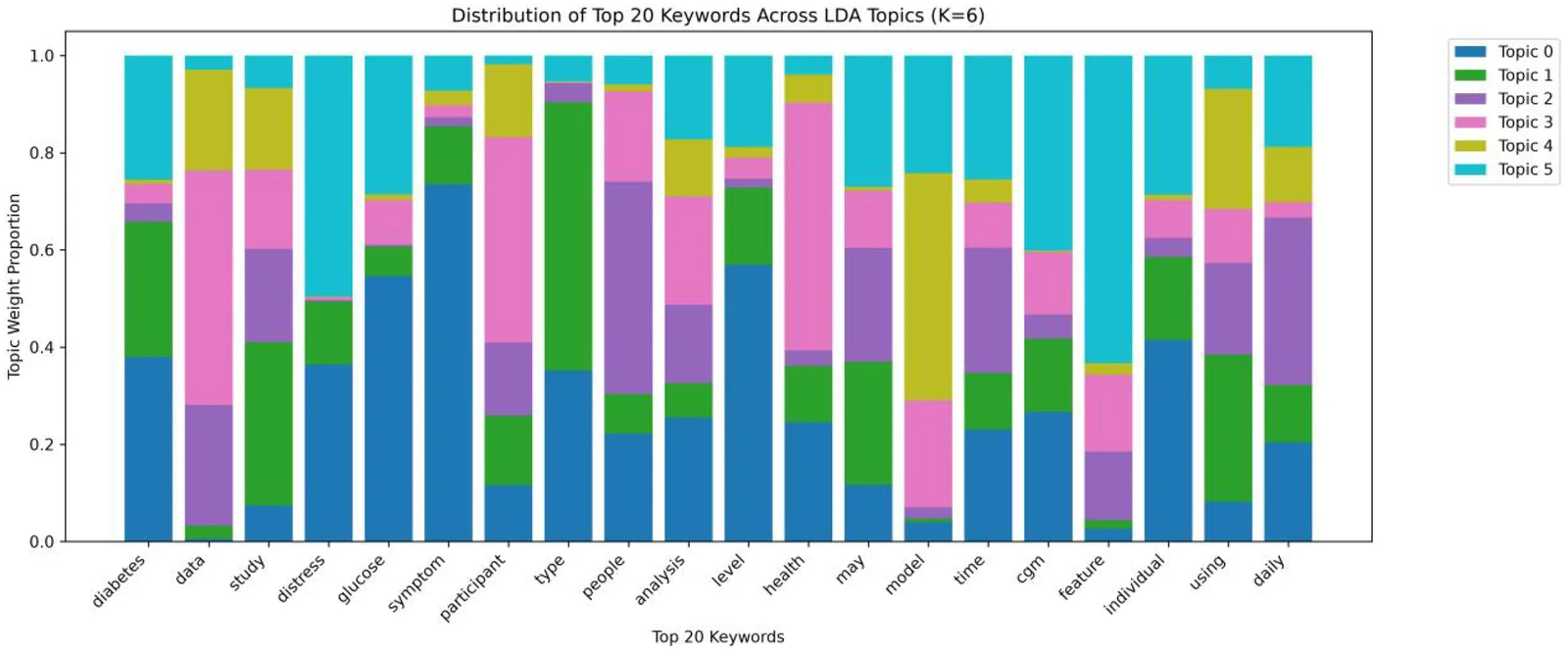
Distribution weight of the top 20 keywords across the six LDA topics.

### 3.4 BERT-based semantic clustering

BERT-based semantic clustering divided the entire corpus (1,354 sentences) into six semantic clusters. The clustering results showed that research in this field mainly focuses on six areas: diabetes distress and mental health (A), mood, sleep and smartphone passive sensing (B), machine learning and model evaluation (C), study design and reporting (D), voice features and stress (E), and glucose, CGM and hypoglycaemia (F) (Figure 5).

**Figure 5:**
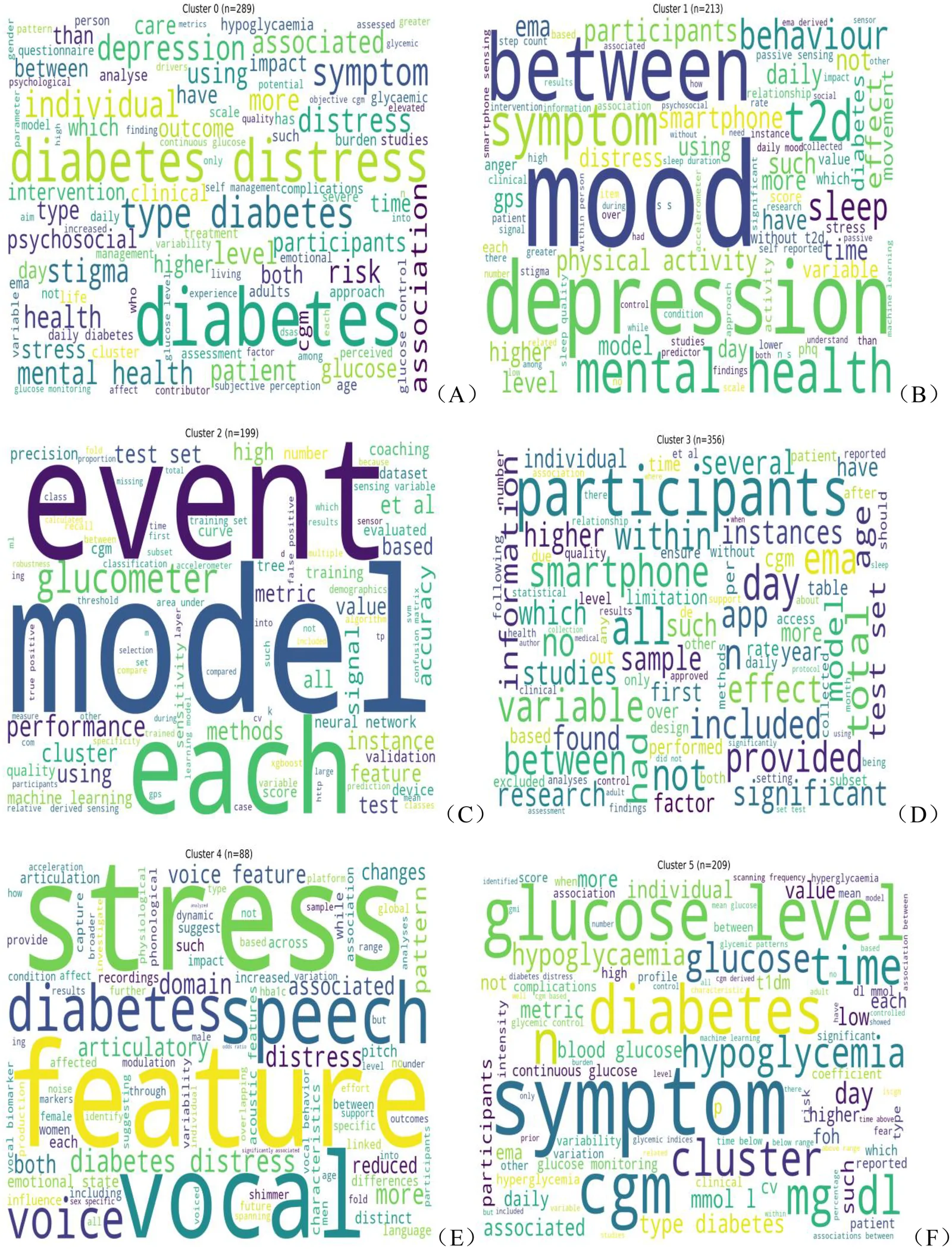
Word clouds for six semantic clusters derived from semantic clustering analysis. Each word cloud represents a semantic cluster, and word size reflects word frequency in that cluster. **(A)** Cluster 0: diabetes distress and mental health; **(B)** Cluster 1: mood, sleep and smartphone passive sensing; **(C)** Cluster 2: machine learning and model evaluation; **(D)** Cluster 3: study design and reporting; **(E)** Cluster 4: voice features and stress; **(F)** Cluster 5: glucose, CGM and hypoglycaemia.

### 3.5 Keyword co-occurrence network analysis

The keyword co-occurrence network exhibited a clear modular organisational structure (Figures 6A,B). In Figure 6A, Signal represents digital phenotyping signals, including four categories: CGM, passive smartphone sensing, voice, and EMA. The core region of the network consisted of digital phenotyping signals and mental health, indicating that the association between digital phenotyping and mental health outcomes is the core issue in current research. Bridging nodes included glucose and machine learning; glucose connected to both Signal and MentalHealth, reflecting that CGM is the main source of digital phenotyping, while machine learning connected to both Signal and MentalHealth, acting as a bridge linking digital phenotyping and outcomes. The peripheral region included the connection between glucose and machine learning, whose co-occurrence frequency was noticeably low. This low co-occurrence frequency may reflect factors such as dictionary construction, sentence segmentation, writing style, or corpus size, and should not be directly interpreted as indicating the extent to which machine learning is applied in modelling glucose data. Figure 6B shows the keyword-level co-occurrence network (Top 30 keywords), which is centred on diabetes, data, model, and mental. The keyword-level co-occurrence network further refined the above structure into four modules: the CGM glucose monitoring module, the diabetes and psychological distress module, the mental health and study design module, and the machine learning modelling module. Bridging nodes connected different modules, suggesting close conceptual associations among digital phenotyping signals, mental health outcomes, and machine learning methods.

**Figure 6:**
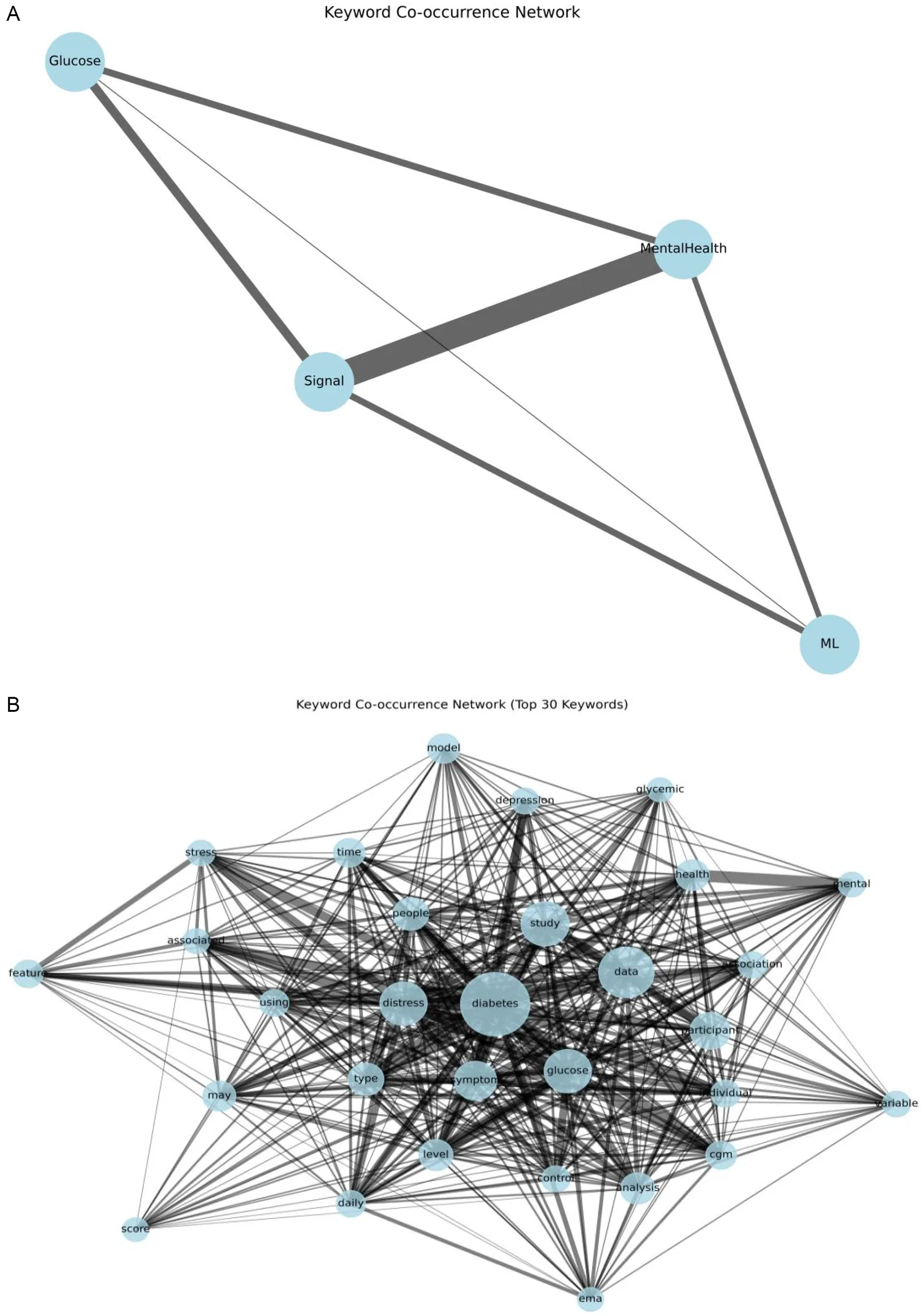
Keyword co-occurrence network. **(A)** Conceptual category network; **(B)** Network of the top 30 frequent keywords.

### 3.6 Co-occurrence pattern analysis

Co-occurrence pattern analysis (Figures 7, 8) showed that the co-occurrence frequency between digital phenotyping signals and mental health outcomes was the highest, indicating that their association is the core issue in current research. The co-occurrence frequencies of glucose with digital phenotyping signals, glucose with mental health, machine learning with digital phenotyping signals, and machine learning with mental health were intermediate, indicating that CGM is the main source of digital phenotyping signals and that machine learning connects both digital phenotyping signals and mental health, acting as a bridge. Notably, the co-occurrence frequency between glucose and machine learning was noticeably low; this low co-occurrence frequency requires cautious interpretation and may be influenced by the dictionary and corpus. In addition, all 10 studies included in this review were monitoring or prediction studies, with no interventional design; therefore, the “monitoring-prediction-intervention” closed-loop design is a direction worthy of attention in current research.

**Figure 7:**
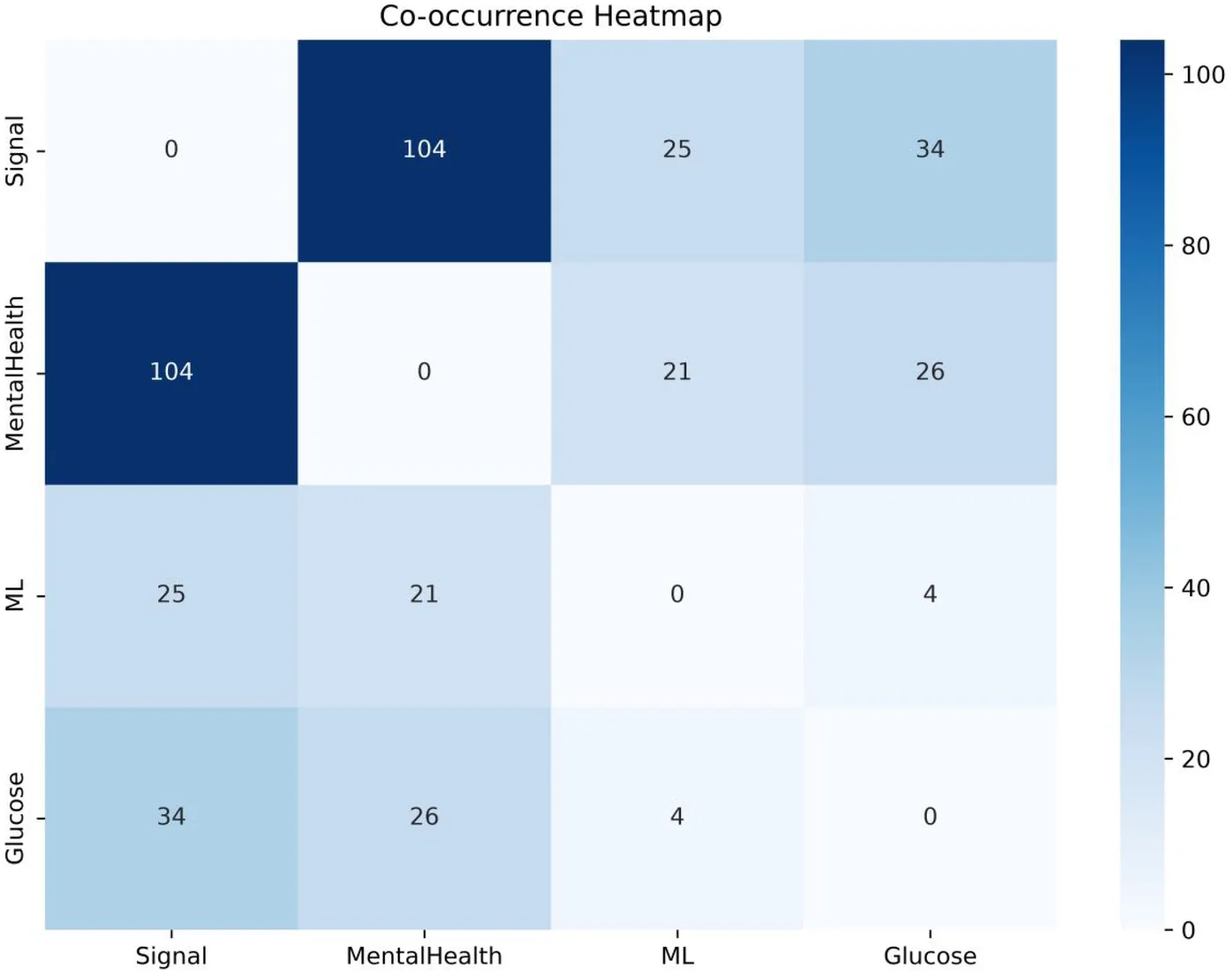
Heatmap showing co-occurrence frequencies among four conceptual categories.

**Figure 8:**
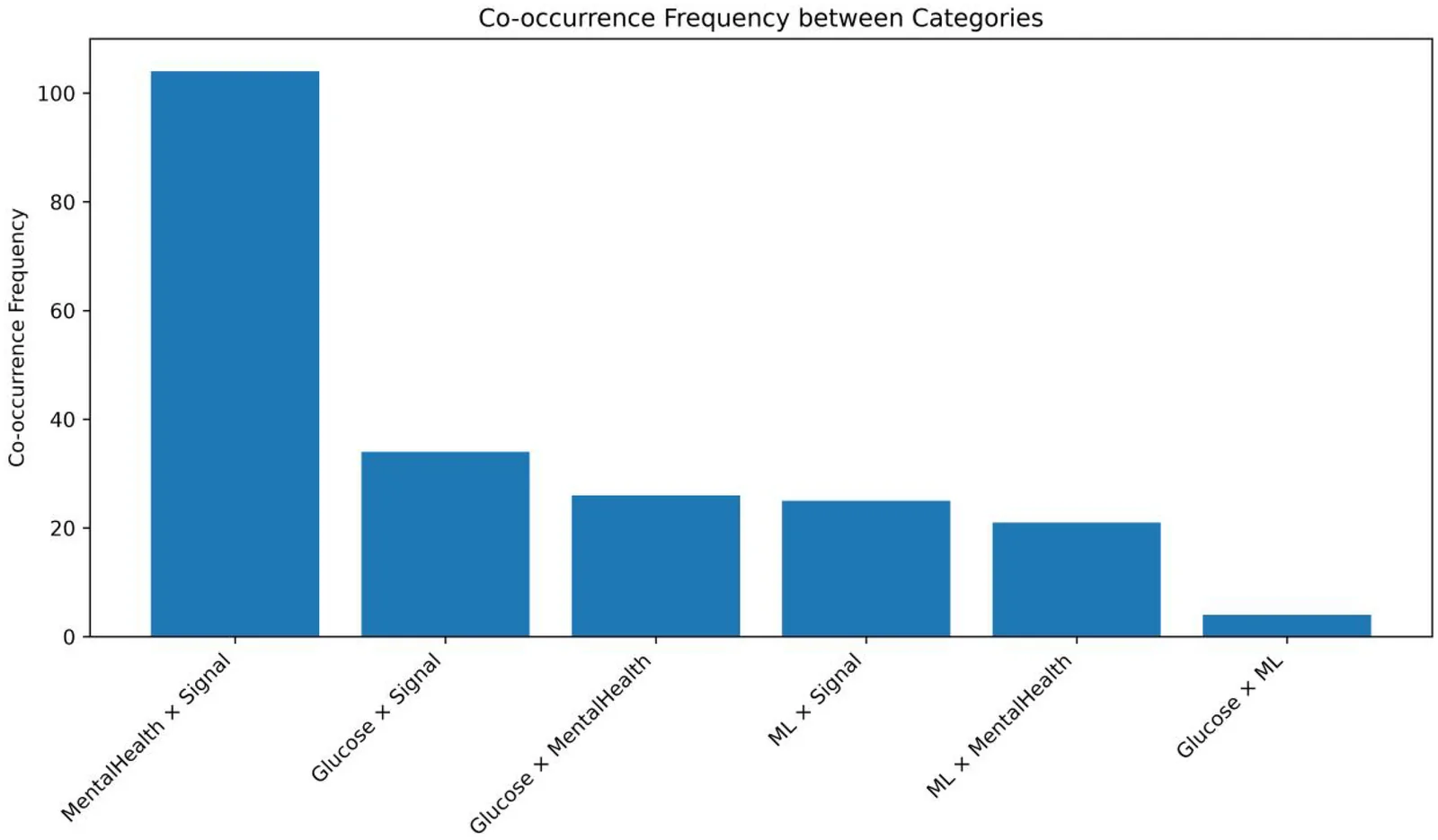
Bar plot of pairwise co-occurrence frequency between conceptual categories.

## 4 Discussion

### 4.1 Main findings on digital phenotyping for mental health monitoring in patients with diabetes

By integrating LDA topic modelling, BERT semantic clustering, and keyword co-occurrence analysis, this review systematically mapped the research evidence on digital phenotyping for mental health monitoring and individualised risk prediction in people with diabetes. Evidence from topic modelling, semantic clustering, and keyword co-occurrence analysis indicated that digital phenotyping does not function merely through isolated signal collection; rather, its value lies in the integrated operation of multiple digital phenotyping signals, such as CGM, passive smartphone sensing, voice features, and ecological momentary assessment. Therefore, the existing evidence suggests that digital phenotyping may function not only through recording or monitoring, but also by transforming signals into information usable for risk identification through machine learning methods, thereby assisting mental health assessment. The association between digital phenotyping signals and mental health outcomes is the core issue in current research. Notably, the co-occurrence frequency between glucose and machine learning was noticeably low. It should be pointed out that this finding may be influenced by the keyword dictionary and sentence segmentation approach, and should not be directly interpreted as reflecting the actual extent of machine learning application in modelling glucose data. In addition, no intervention studies were reported among the included studies. Taken together, the above evidence suggests that digital phenotyping may provide a more continuous, objective, and individualised pathway for mental health monitoring and prediction in people with diabetes.

### 4.2 Associations between digital phenotyping signals and mental health outcomes

The core signal types identified in this review are broadly consistent with theories of mental health monitoring. Through multiple digital phenotyping signals collected in real time, passively or actively—CGM, passive smartphone sensing, voice features, and ecological momentary assessment (EMA)—digital phenotyping supports continuous self-observation, emotion recognition, and mental health risk prediction. CGM was the most important digital phenotyping signal. One included study suggested that the association between subjectively perceived glucose control and diabetes distress was stronger than that of objective CGM indicators (15), suggesting that future interventions should simultaneously attend to patients' subjective interpretation of glucose. Voice features may help distinguish between stress and diabetes-related psychological distress, suggesting that voice biomarkers may become low-cost, non-invasive tools for mental health monitoring, and that smartphone-based voice analysis may serve as a scalable, non-invasive biosignal for continuous monitoring (20). Passive smartphone sensing (accelerometer, GPS, screen time, call logs) was associated with depressive symptoms and daily mood, and depressive symptoms were significantly associated with low social contact and low daytime activity (12). Taken together, these findings suggest that digital phenotyping signals encompass not only glucose-related physiological indicators but also multiple dimensions such as behaviour, voice, and emotion, providing a multidimensional signal basis for real-time monitoring and individualised risk prediction of mental health in people with diabetes.

### 4.3 Application of machine learning methods in digital phenotyping modeling

The machine learning methods identified in this review encompass traditional algorithms (random forest, SVM, naive Bayes, XGBoost, LightGBM, neural networks) and deep learning methods. Interpretability tools such as SHAP can provide clinicians with understandable predictive rationales, thereby improving the acceptability of models in clinical settings;In diabetes prediction, they can simultaneously achieve relatively high predictive performance and clinical transparency (22). However, different studies were highly heterogeneous in terms of prediction targets, algorithm selection, sample size, validation strategies, performance metrics, and interpretability methods; a detailed comparison is provided in Supplementary Material S5. Model performance cannot be directly compared across studies because populations, outcomes, datasets, and validation strategies differ. In addition, most studies did not report calibration, class imbalance handling, or missing data handling methods; future studies should improve the transparency of reporting these methodological details. This review also found that external validation of models was generally lacking; the absence of external validation is a major limitation, and multicentre validation is also lacking. This suggests that future studies should strengthen multicentre, large-sample external validation to improve model generalisability. Notably, the application of explainable artificial intelligence methods is becoming a trend and may help narrow the gap between algorithmic performance and clinical applicability (23).

### 4.4 Research gaps and future directions

Based on the results of this review, we identified the following main research gaps. First, this review found that three studies conducted risk prediction modelling (12, 13, 17), and only three studies genuinely achieved individualised risk prediction: Ehrmann et al. (15) and Hermanns et al. (16) used *n*-of-1 analysis to build separate glucose-psychological state association models for each patient, and Lee et al. (19) used a Gaussian mixture model to identify four glycaemic pattern subgroups, which constitutes subgroup prediction. The remaining four studies were purely association analyses. Existing evidence is mainly concentrated at the monitoring and prediction levels, no intervention studies have been reported, and a complete “monitoring-identification-prediction-intervention-evaluation” loop has not yet been formed. Future research needs to explore how to translate risk assessment results into actionable intervention strategies on the basis of prediction model validation. Second, comorbidity-oriented modelling remains insufficient. Most studies focused on a single outcome and rarely simultaneously considered comorbid states such as depression, anxiety, and diabetes-related distress. Future research should conduct comorbidity-oriented modelling and explore the interaction patterns among different psychological states. In addition, population coverage is uneven: samples were mainly young and middle-aged adults, and research on older adults with diabetes is clearly insufficient. Future research should expand population coverage, with particular attention to older adults with diabetes and populations with low digital health literacy. Furthermore, most existing studies came from a single country or a single centre, lacking cross-national multicentre collaboration and external validation. The 10 included studies came from Japan, South Korea, Germany, Ireland, Luxembourg, the United States, and India, respectively, but all used single-centre designs, and no multicentre, cross-national collaborative studies or externally validated models were identified. Future research should strengthen multicentre, cross-national external validation to improve model generalisability. Finally, the application of physiological signals such as voice and heart rate variability is limited. Among the studies included in this review, only one explored voice features, and no study explored autonomic nervous system signals such as heart rate variability or electrodermal activity. Future research should expand the spectrum of digital phenotyping signals to improve the multimodal precision of mental health monitoring.

### 4.5 Limitations

This review has the following limitations. First, the number of included studies was limited (only 10), and the study designs were mainly cross-sectional and observational, which limits the generalisability of the conclusions. The sample sizes of the included studies varied widely (47 to 142, 432); some were small-sample feasibility studies, while others were based on large retrospective datasets, and there was high heterogeneity in sample size, study design, follow-up duration, and dataset scale. Second, the corpus size was small (1,354 sentences in total), and sentence-level co-occurrence and clustering analyses may overestimate the weight of longer documents and may not fully represent study-level evidence. Future research should perform document-level sensitivity analyses to verify the robustness of sentence-level analysis results. Third, the silhouette scores of BERT semantic clustering were low across multiple *K* values (all <0.07), indicating limited separation between clusters and a high degree of overlap among research themes. In addition, the independently optimal K for BERT clustering was *K* = 2 (silhouette score = 0.0656); *K* = 6 was ultimately selected for comparison with the LDA topic model rather than because it was independently optimal for BERT. Fourth, the LDA topic stability assessment showed that the *K* = 6 topic structure exhibited moderate stability across five independent runs (mean Jaccard similarity = 0.3354), with some topic boundaries fluctuating, suggesting that LDA topic division may be affected by random initialisation. Fifth, the keyword dictionary was predefined, which may introduce analyst bias. In addition, no synonym merging was applied during the co-occurrence analysis: concepts expressed under multiple surface forms (e.g., depression and depressive) were counted as separate entries, and this conservative strategy may have led to an underestimation of certain co-occurrence frequencies. Only English and Chinese literature was included, which may introduce selection bias. Sixth, no formal risk of bias assessment was performed, consistent with the scoping review methodological framework, but descriptive methodological indicators such as study design and sample size were extracted to aid interpretation of evidence quality. Seventh, all included studies were monitoring or prediction studies, with no interventional design; therefore, intervention-related variables were not included during data extraction. This limitation reflects the current evidence characteristics of the field rather than a flaw of the review itself.

## 5 Conclusions

This article explored the application of digital phenotyping signals in mental health monitoring and individualised risk prediction through machine learning for people with diabetes. Through a scoping review, it mapped signal types, machine learning methods, mental health outcomes, and co-occurrence patterns in digital phenotyping for mental health monitoring in people with diabetes. Preliminary evidence suggests that digital phenotyping is associated with mental health outcomes in people with diabetes, and that machine learning methods show some potential in exploratory predictive modelling. However, the current evidence is limited to the association and monitoring levels and is insufficient to support inferences about causality or clinical intervention effects. Future research should, through prospective validation and clinical utility assessment, improve multimodal signal integration, and should focus on closed-loop monitoring-prediction-intervention design, multimodal signal integration, external validation of models, user usability, acceptability, privacy protection, health equity, and intervention studies. Current evidence is limited to the monitoring and prediction levels and does not yet support the claim that digital phenotyping is already capable of clinical intervention or clinical implementation; future research should focus on prospective validation, clinical utility assessment, intervention studies, and multicentre cross-national collaboration, rather than directly asserting their clinical usability.

## Data Availability

The original contributions presented in the study are included in the article/Supplementary Material, further inquiries can be directed to the corresponding author/s.
