# Supplementary material for: Digital phenotyping for mental health monitoring and personalized prediction in people with diabetes: a Python-based scoping review
Source: Front Digit Health. 2026 Oct 28;8:2005262. doi: 10.3389/fdgth.2026.2005262 (PMC13751931; doi:10.3389/fdgth.2026.2005262)
Supplement: Supplementary file 1 [file Table1.doc]

| **Database** | **Platform** | **Searchdate** | **Searchfields** | **Datelimit** | **Results** |
| --- | --- | --- | --- | --- | --- |
| PubMed | NCBI | 2026-09-10 | Title/Abstract + MeSH | Inception–2026-09-10 | 307 |
| Embase | Elsevier | 2026-09-10 | ti,ab,kw + Emtree | Inception–2026-09-10 | 332 |
| CINAHL | EBSCO | 2026-09-10 | TI/AB + MH | Inception–2026-09-10 | 167 |
| Web of Science | Clarivate | 2026-09-10 | TS | Inception–2026-09-10 | 252 |
| Scopus | Elsevier | 2026-09-10 | TITLE-ABS-KEY | Inception–2026-09-10 | 228 |
| Cochrane Library | Wiley | 2026-09-10 | ti,ab,kw + MeSH | Inception–2026-09-10 | 441 |
| CNKI | CNKI | 2026-09-10 | Topic | Inception–2026-09-10 | 111 |
| 万方 | Wanfang | 2026-09-10 | Topic | Inception–2026-09-10 | 874 |
| 维普 | VIP | 2026-09-10 | All Fields | Inception–2026-09-10 | 164 |
| CBM | CBM | 2026-09-10 | Common Fields | Inception–2026-09-10 | 92 |

Pubemd

(("Diabetes Mellitus"[Mesh] OR "Diabetes Mellitus, Type 1"[Mesh] OR "Diabetes Mellitus, Type 2"[Mesh] OR "type 1 diabetes"[Title/Abstract] OR "type 2 diabetes"[Title/Abstract]) AND ("digital phenotyping"[Title/Abstract] OR smartphone*[Title/Abstract] OR "wearable devices"[Mesh] OR "smart bracelet"[Title/Abstract] OR "passive monitoring"[Title/Abstract] OR "behavioral data"[Title/Abstract] OR sensor*[Title/Abstract] OR "digital biomarker*"[Title/Abstract] OR "machine learning"[Title/Abstract] OR "artificial intelligence"[Title/Abstract])) AND ("psychological distress"[Title/Abstract] OR "diabetes distress"[Title/Abstract] OR anxiety [Title/Abstract] OR depress*[Title/Abstract] OR fear [Title/Abstract] OR "mental health"[Title/Abstract] OR psychology [Title/Abstract])

CNKI

主题:(糖尿病 OR 1型糖尿病 OR 2型糖尿病) and 主题:(心理健康 OR 心理痛苦 OR 焦虑 OR 抑郁 OR 恐惧 OR 心理 OR 糖尿病痛苦) and 主题:(数字表型 OR 手机 OR 可穿戴设备 OR 智能手环 OR 被动监测 OR 行为数据 OR 传感器 OR 数字生物标志物 OR 人工智能 OR 机器学习)

WanFang

主题:(糖尿病 OR 1型糖尿病 OR 2型糖尿病) and 主题:(心理健康 OR 心理痛苦 OR 焦虑 OR 抑郁 OR 恐惧 OR 心理 OR 糖尿病痛苦) and 主题:(数字表型 OR 手机 OR 可穿戴设备 OR 智能手环 OR 被动监测 OR 行为数据 OR 传感器 OR 数字生物标志物 OR 人工智能 OR 机器学习)

VIP

[((((任意字段=糖尿病 OR 任意字段=1型糖尿病) OR 任意字段=2型糖尿病) AND ((((((任意字段=心理健康 OR 任意字段=心理痛苦) OR 任意字段=焦虑) OR 任意字段=抑郁) OR 任意字段=恐惧) OR 任意字段=心理) OR 任意字段=糖尿病痛苦)) AND (((((((((任意字段=数字表型 OR 任意字段=手机) OR 任意字段=可穿戴设备) OR 任意字段=智能手环) OR 任意字段=被动监测) OR 任意字段=行为数据) OR 任意字段=传感器) OR 任意字段=数字生物标志物) OR 任意字段=人工智能) OR 任意字段=机器学习))](https://qikan.cqvip.com/Qikan/search/index?LngMySearHistoryIdGuid=cd680553-6b63-4e52-bb22-99b7e01d7db5&from=Qikan_Article_History)

CBM

[( "糖尿病"[常用字段:智能] OR "1型糖尿病"[常用字段:智能] OR "2型糖尿病"[常用字段:智能]) AND( "心理健康"[常用字段:智能] OR "心理"[常用字段:智能] OR "焦虑"[常用字段:智能] OR "抑郁"[常用字段:智能] OR "恐惧"[常用字段:智能] OR "糖尿病痛苦"[常用字段:智能] OR "心理痛苦"[常用字段:智能]) AND( "数字表型"[常用字段:智能] OR "手机"[常用字段:智能] OR "可穿戴设备"[常用字段:智能] OR "智能手环"[常用字段:智能] OR "被动监测"[常用字段:智能] OR "传感数据"[常用字段:智能] OR "传感器"[常用字段:智能] OR "行为数据"[常用字段:智能] OR "数字生物标志物"[常用字段:智能] OR "人工智能"[常用字段:智能] OR "机器学习"[常用字段:智能])](javascript:toDoRelimitSearch();)

Web of science

TS=("psychological distress" OR "diabetes distress" OR anxiety OR depress* OR fear OR "mental health" OR psychology)ANDTS=("digital phenotyping" OR smartphone* OR "wearable devices" OR "smart bracelet" OR "passive monitoring" OR "behavioral data" OR sensor* OR "digital biomarker*" OR "machine learning" OR "artificial intelligence")ANDTS=("diabetes mellitus" OR "type 1 diabetes" OR "type 2 diabetes")AND(DT=("Article" OR "Early Access" OR "Editorial Material"))AND(OA=("Open Access"))

CINAHL

(MH "Diabetes Mellitus, Type 1+" OR MH "Diabetes Mellitus, Type 2+" OR "type 1 diabetes" OR "type 2 diabetes" OR diabetes)AND(MH "Anxiety+" OR MH "Depression+" OR MH "Psychological Distress" OR "psychological distress" OR "diabetes distress" OR anxiety OR depress*)AND(MH "Wearable Electronic Devices" OR "digital phenotyping" OR smartphone* OR "wearable devices" OR "smart bracelet" OR "passive monitoring" OR "behavioral data" OR sensor* OR "digital biomarker*" OR "machine learning" OR "artificial intelligence")

Scoups

TITLE-ABS-KEY (("diabetes distress" OR "psychological distress" OR anxiety OR depress*))AND TITLE-ABS-KEY (("digital phenotyping" OR "passive monitoring" OR "wearable devices" OR sensor* OR "machine learning" OR "artificial intelligence" OR "behavioral data"))AND TITLE-ABS-KEY (("type 1 diabetes" OR "type 2 diabetes"))AND NOT TITLE-ABS-KEY (animal* OR mouse OR rat OR murine OR rabbit OR dog)

Cochrane Liabrary

#1 - (("psychological distress" OR "diabetes distress" OR anxiety OR depress* OR fear OR "mental health" OR psychology):ti,ab,kw AND ("digital phenotyping" OR smartphone* OR "wearable devices" OR "smart bracelet" OR "passive monitoring" OR "behavioral data" OR sensor* OR "digital biomarker*" OR "machine learning" OR "artificial intelligence"):ti,ab,kw AND ("diabetes mellitus" OR "type 1 diabetes" OR "type 2 diabetes"):ti,ab,kw NOT (animal* OR mouse OR rat OR murine OR rabbit OR dog):ti,ab,kw)

Embase

("psychological distress" OR "diabetes distress" OR anxiety OR depress* OR fear OR "mental health" OR psychology):ti,ab,kw AND ("digital phenotyping" OR smartphone* OR "wearable devices" OR "smart bracelet" OR "passive monitoring" OR "behavioral data" OR sensor* OR "digital biomarker*" OR "machine learning" OR "artificial intelligence"):ti,ab,kw AND ("diabetes mellitus" OR "type 1 diabetes" OR "type 2 diabetes"):ti,ab,kw NOT (animal* OR mouse OR rat):ti,ab,kw
