# Supplementary material for: Digital phenotyping for mental health monitoring and personalized prediction in people with diabetes: a Python-based scoping review
Source: Front Digit Health. 2026 Oct 28;8:2005262. doi: 10.3389/fdgth.2026.2005262 (PMC13751931; doi:10.3389/fdgth.2026.2005262)
Supplement: Supplementary file 2 [file Table2.doc]

**Table S2. PCC mapping of included studies.**

| **Study** | **Population (P)** | **Concept (C): Digital phenotyping signal** | **Context (C): Mental health outcome** | **Eligibility justification** |
| --- | --- | --- | --- | --- |
| Sarda et al., 2019 [12] | Type 1 + Type 2 diabetes | Smartphone passive sensing (accelerometer, GPS, ambient light, call log) | Depression (PHQ-9) | Meets P (diabetes), C (passive sensing), C (mental health outcome) |
| Yu et al., 2021 [13] | Type 1 + Type 2 diabetes | Glucometer, app interaction, coaching interaction | Mental health risk (prescription/claims) | Meets P, C, C |
| Hohendorff et al., 2022 [14] | Type 1 diabetes | isCGM (FreeStyle Libre) | Fear of hypoglycemia (HFS-II) | Meets P, C, C |
| Ehrmann et al., 2024 [15] | Type 1 + Type 2 diabetes | CGM + EMA | Diabetes distress (PAID), depression (CES-D) | Meets P, C, C |
| Hermanns et al., 2025 [16] | Type 1 + Type 2 diabetes | CGM + EMA | Depression (CES-D), diabetes distress (PAID) | Meets P, C, C |
| Sakane et al., 2026 [17] | Type 1 diabetes | CGM (FreeStyle Libre) | Diabetes distress (PAID) | Meets P, C, C |
| McInerney et al., 2026 [18] | Type 2 diabetes | Smartphone (accelerometer, GPS, EMA) | Daily mood (happy, sad, stress, anger) | Meets P, C, C |
| Lee et al., 2026 [19] | Type 2 diabetes | CGM (FreeStyle Libre Pro) | Diabetes distress (DDS-17) | Meets P, C, C |
| Bour et al., 2026 [20] | Type 1 + Type 2 diabetes | Voice (DisVoice) | Stress, diabetes distress (PAID) | Meets P, C, C |
| Kim et al., 2026 [21] | Type 1 diabetes | CGM (Guardian Connect, Dexcom, FreeStyle Libre) | Stigma (DSAS-1), anxiety (GAD-7), depression (PHQ-9), diabetes distress (DDS) | Meets P, C, C |
