# Supplementary material for: Digital phenotyping for mental health monitoring and personalized prediction in people with diabetes: a Python-based scoping review
Source: Front Digit Health. 2026 Oct 28;8:2005262. doi: 10.3389/fdgth.2026.2005262 (PMC13751931; doi:10.3389/fdgth.2026.2005262)
Supplement: Supplementary file 3 [file Table3.doc]

# **Supplementary Material S3: Keyword Dictionary and Synonym Mapping Rules**

## **Table S3.1 Complete keyword dictionary for four concept categories**

| **Category** | **Keywords included** |
| --- | --- |
| **Signal** | cgm, continuous glucose monitoring, smartphone, wearable, accelerometer, gps, voice, ema, ecological momentary assessment, passive sensing, iscgm, rtcgm |
| **MentalHealth** | depression, depressive, anxiety, diabetes distress, psychological distress, phq, gad, paid, dds, mental health, fear of hypoglycemia, foh |
| **ML** | machine learning, xgboost, lightgbm, neural network, shap, clustering, random forest, svm, prediction, python |
| **Glucose** | hba1c, time in range, tir, hypoglycemia, hyperglycemia, glucose variability |

## **Table S3.2 Handling of synonyms and abbreviations**

To make the keyword-matching rule explicit and reproducible, the following four rules were applied during sentence-level co-occurrence analysis:

| **Rule** | **Handling** |
| --- | --- |
| **(i) Synonyms** | Principal surface forms were listed separately in the dictionary (e.g., depression and depressive; anxiety and anxious; diabetes distress and psychological distress; machine learning and deep learning). Each listed form was matched independently by exact string matching after lowercasing. No automatic synonym merging or semantic normalisation was applied. |
| **(ii) Abbreviations** | Common abbreviations (e.g., cgm, ema, iscgm, rtcgm, phq, gad, paid, dds, foh, tir, hba1c) were retained as distinct dictionary entries alongside their full forms. Both the abbreviation and the full form were included so that either surface form could be matched. |
| **(iii) Multi-word expressions** | Multi-word terms (e.g., continuous glucose monitoring, ecological momentary assessment, machine learning) were matched as contiguous phrases. |
| **(iv) Morphological variants and negation** | No stemming or lemmatisation was applied during dictionary matching (lemmatisation was used only during text preprocessing for word-frequency analysis, as described in Section 2.7.1). No negation detection or semantic reversal was performed; sentences were processed in their original word order. |

**Note:**The predefined keyword dictionary was applied directly to the sentence-level corpus using exact string matching after lowercasing. All terms were converted to lowercase before matching. This conservative matching strategy was chosen to preserve the original terminology of the source literature and to avoid arbitrary merging decisions. We acknowledge that this approach may underestimate co-occurrence counts for concepts that appear in multiple surface forms (for example, depression and depressive were counted as two separate entries). This limitation has been added to the Limitations section (Section 4.5) of the manuscript.

## **Table S3.3 Potential effect of not performing synonym merging**

| **Concept** | Dictionary entries used | Merged during analysis? | Potential effect on co-occurrence counts |
| --- | --- | --- | --- |
| **Depression** | depression, depressive | No | May be split across two entries |
| **Anxiety** | anxiety, phq, gad | No | May be split across three entries |
| **Diabetes distress** | diabetes distress, psychological distress, paid, dds | No | May be split across four entries |
| **CGM** | cgm, continuous glucose monitoring, iscgm, rtcgm | No | May be split across four entries |
| **Machine learning** | machine learning, xgboost, lightgbm, neural network, shap, random forest, svm, clustering, prediction, python | No | May be split across multiple entries |

**Note:** The absence of synonym merging is a deliberate methodological choice, and its potential effect on the reported co-occurrence frequencies is acknowledged as a limitation. Future studies could apply explicit synonym normalisation and compare results.
