# Supplementary material for: Digital phenotyping for mental health monitoring and personalized prediction in people with diabetes: a Python-based scoping review
Source: Front Digit Health. 2026 Oct 28;8:2005262. doi: 10.3389/fdgth.2026.2005262 (PMC13751931; doi:10.3389/fdgth.2026.2005262)
Supplement: Supplementary file 4 [file Table4.doc]

**Supplementary Material S4: BERT Clustering Results Across Different K Values**

**Table S4. BERT K-Means clustering results for K = 2 to 10.**

| **Number of clusters (K)** | **SSE** | **Silhouette score** |
| --- | --- | --- |
| 2 | 999.80 | 0.0656 |
| 3 | 972.52 | 0.0545 |
| 4 | 948.16 | 0.0572 |
| 5 | 933.42 | 0.0603 |
| **6** | **916.83** | **0.0382** |
| 7 | 901.63 | 0.0400 |
| 8 | 891.72 | 0.0410 |
| 9 | 882.81 | 0.0420 |
| 10 | 875.87 | 0.0445 |

The independent optimal K based on the highest silhouette score is K=2 (silhouette = 0.0656). However, K=6 was selected for direct comparison with the LDA topic model (K=6), rather than because K=6 was the independently optimal number of clusters. This limitation has been explicitly acknowledged in the Limitations section of the manuscript.

Note: The red dashed line indicates the selected number of clusters (K = 6). SSE = sum of squared errors.
