# Supplementary material for: Digital phenotyping for mental health monitoring and personalized prediction in people with diabetes: a Python-based scoping review
Source: Front Digit Health. 2026 Oct 28;8:2005262. doi: 10.3389/fdgth.2026.2005262 (PMC13751931; doi:10.3389/fdgth.2026.2005262)
Supplement: Supplementary file 5 [file Table5.doc]

**Table S5. Comparison of machine learning models in included studies.**

| Study | Prediction target | Algorithm | Sample size | Validation strategy | Key performance | Explainability |
| --- | --- | --- | --- | --- | --- | --- |
| Sarda et al., 2019 [12] | Depression (PHQ-9) | XGBoost, Random Forest, SVM, Decision Tree, AdaBoost | 47 | Train/test split | Accuracy: XGBoost 81.1%, RF 80.0% | Not reported |
| Yu et al., 2021 [13] | Mental health risk (prescription/claims) | LightGBM ensemble (10 models) + SHAP | 142，432 | 3 test sets | AUC > 0.65 in all test sets | SHAP |
| Hohendorff et al., 2022 [14] | Fear of hypoglycemia (HFS-II) | Correlation analysis, multiple regression | 77 | Not applicable | r = -0.25 (scanning frequency vs HFS-II) | Not applicable |
| Ehrmann et al., 2024 [15] | Diabetes distress (PAID), depression (CES-D) | Bayesian linear mixed-effects + N-of-1 | 379 | N-of-1 | Individual drivers predicted 3-month outcomes | Not applicable |
| Hermanns et al., 2025 [16] | Depression (CES-D), diabetes distress (PAID) | Linear mixed-effects + N-of-1 | 371 | N-of-1 | 8 symptoms associated with glucose; depression/distress increased symptom scores up to 12% | Not applicable |
| Sakane et al., 2026 [17] | Diabetes distress (PAID) | Neural network, RF, SVM, Naive Bayes, k-NN, XGBoost, LightGBM (10 algorithms) | 259 | Cross-validation | Neural network best: AUC = 0.728, accuracy = 0.744 | Feature importance (LBGI most important) |
| McInerney et al., 2026 [18] | Daily mood (happy, sad, stress, anger) | Dynamic structural equation model (DSEM) | 61 | Not applicable | Sleep quality and physical activity associated with daily mood | Not applicable |
| Lee et al., 2026 [19] | Diabetes distress (DDS-17) | Gaussian Mixture Model (GMM) | 137 | Not reported | 4 glycemic patterns identified; persistent hyperglycemia group had highest distress (mean = 2.37) | Not reported |
| Bour et al., 2026 [20] | Stress, diabetes distress (PAID) | Multivariate ordinal logistic regression | 679 | Not reported | Stress associated with 25 voice features; distress with 18 features | Not applicable |
| Kim et al., 2026 [21] | Stigma (DSAS-1), anxiety (GAD-7), depression (PHQ-9), distress (DDS) | Linear regression, logistic regression | 104 | Not reported | Stigma associated with TBR < 3.0 mmol/L; stigma and anxiety OR = 1.086 | Not applicable |

**Note:** AUC = area under the receiver operating characteristic curve; PHQ-9 = Patient Health Questionnaire-9; GAD-7 = Generalized Anxiety Disorder-7; PAID = Problem Areas in Diabetes; CES-D = Center for Epidemiologic Studies Depression Scale; HFS-II = Hypoglycemia Fear Survey-II; DSAS-1 = Type 1 Diabetes Stigma Assessment Scale; DDS = Diabetes Distress Scale; TBR = time below range; LBGI = Low Blood Glucose Index; SHAP = SHapley Additive exPlanations. Model performance should not be directly compared across studies due to differences in populations, outcomes, datasets, and validation strategies.
